# Exploring variation in low-value care: a multilevel modelling study

**DOI:** 10.1186/s12913-019-4159-1

**Published:** 2019-05-30

**Authors:** Tim Badgery-Parker, Yingyu Feng, Sallie-Anne Pearson, Jean-Frederic Levesque, Susan Dunn, Adam G. Elshaug

**Affiliations:** 1 Faculty of Medicine and Health, School of Public Health, Menzies Centre for Health Policy, Charles Perkins Centre, The University of Sydney, Level 2, Charles Perkins Centre D17, Sydney, NSW 2006 Australia; 2 Centre for Big Data Research in Health, University of New South Wales, Sydney, Australia; 3 Agency for Clinical Innovation, Sydney, NSW Australia; 4 NSW Ministry of Health, Sydney, Australia

**Keywords:** Low-value care, Multilevel logistic regression, Choosing wisely, Disinvestment

## Abstract

**Background:**

Whether patients receive low-value hospital care (care that is not expected to provide a net benefit) may be influenced by unmeasured factors at the hospital they attend or the hospital’s Local Health District (LHD), or the patients’ areas of residence. Multilevel modelling presents a method to examine the effects of these different levels simultaneously and assess their relative importance to the outcome. Knowing which of these levels has the greatest contextual effects can help target further investigation or initiatives to reduce low-value care.

**Methods:**

We conducted multilevel logistic regression modelling for nine low-value hospital procedures. We fit a series of six models for each procedure. The baseline model included only episode-level variables with no multilevel structure. We then added each level (hospital, LHD, Statistical Local Area [SLA] of residence) separately and used the change in the *c* statistic from the baseline model as a measure of the contribution of the level to the outcome. We then examined the variance partition coefficients (VPCs) and median odds ratios for a model including all three levels. Finally, we added level-specific covariates to examine if they were associated with the outcome.

**Results:**

Analysis of the *c* statistics showed that hospital was more important than LHD or SLA in explaining whether patients receive low-value care. The greatest increases were 0.16 for endoscopy for dyspepsia, 0.13 for colonoscopy for constipation, and 0.14 for sentinel lymph node biopsy for early melanoma. SLA gave a small increase in *c* compared with the baseline model, but no increase over the model with hospital. The VPCs indicated that hospital accounted for most of the variation not explained by the episode-level variables, reaching 36.8% (95% CI, 31.9–39.0) for knee arthroscopy. ERCP (8.5%; 95% CI, 3.9–14.7) and EVAR (7.8%; 95% CI, 2.9–15.8) had the lowest residual variation at the hospital level. The variables at the hospital, LHD and SLA levels that were available for this study generally showed no significant effect.

**Conclusions:**

Investigations into the causes of low-value care and initiatives to reduce low-value care might best be targeted at the hospital level, as the high variation at this level suggests the greatest potential to reduce low-value care.

**Electronic supplementary material:**

The online version of this article (10.1186/s12913-019-4159-1) contains supplementary material, which is available to authorized users.

## Background

Knowing where low-value care (tests and interventions for which the benefit is not expected to outweigh the harm and/or costs [1]) occurs and factors associated with low-value care is important in planning to reduce low-value care. Factors at various levels can affect whether a patient receives low-value care. For example, hospitals vary in their rates of low-value care [2, 3], so the risk of a patient receiving low-value care may be affected by which hospital they attend. In New South Wales (NSW, the most populous state of Australia), hospitals belong to Local Health Districts (LHDs), which govern funding and can make policy for all hospitals in the LHD. Thus, the LHD in which a patient is treated could influence the care received. Geographic variation analysis has shown that rates of selected services vary by patients’ area of residence [4], and rates of low-value care might also show such geographic variation. Furthermore, whether patients receive appropriate care for lung cancer depends on the distance from the patient’s residence to a specialist hospital [5], and it is similarly possible that whether patients receive low-value care depends on where they live. Understanding the relative importance of hospital, LHD, and area of residence will be useful for guiding programs to reduce low-value care. This information might also provide indirect information about possible pathways that lead to low-value care. For example, variation between hospitals may reflect the treatment preferences of surgeons who operate in the different hospitals, whereas variation between areas of residence may reflect the decisions of general practitioners (GPs) and their referral pathways (in Australia GPs are ‘gate-keepers’ who must refer patients to specialists, including surgeons).

Multilevel modelling presents a statistical method that can model the effects of different levels simultaneously [6, 7]. Such models can estimate the variation between units at different levels (e.g. hospital, LHD, area of residence) and partition the total unexplained variation to the different levels, reflecting their relative importance to the outcome variable. To date, this method has not been used to examine low-value care. Variation in rates of low-value care between hospitals has been reported in Canada [2] and Australia [3], and geographic variation across hospital referral regions has been examined in the United States [8], but no analysis to date has considered all these levels simultaneously to reveal their relative importance.

We have developed measures for low-value inpatient procedures in public hospitals based on administrative data [3, 9]. In this study, we used multilevel modelling for nine of these measures to explore the contributions of hospital, LHD of hospital, and Statistical Local Area (SLA) of patient’s residence in explaining variation in rates of low-value care.

## Methods

### Setting and data

NSW is the most populous state of Australia. Inpatient care is provided at 225 public hospitals in 15 LHDs. We used public hospital admitted patient data from the NSW Health Information Exchange data warehouse for the period 1 July 2010 to 30 June 2017. The data include patient demographic information (e.g. age, sex, area of residence), clinical information (e.g. procedures performed and diagnoses assigned), and administrative information (e.g. private or public patient, emergency or elective care) for every inpatient episode at a public hospital in NSW. The data is organised into hospital ‘stays’ which comprise one or more ‘episodes’. An episode ends when the type of care changes (e.g. from acute to rehabilitation) or the patient is transferred or discharged.

We used the Australian Bureau of Statistics Statistical Local Areas (2011) to define the patients’ areas of residence [10]. In NSW, there are 199 SLAs, with estimated residential populations ranging from about 400 to about 155,000 (median, approximately 23,000).

We downloaded Index of Relative Socioeconomic Advantage and Disadvantage (IRSAD) [11], Remoteness Categories [12], and Estimated Residential Populations from the Australian Bureau of Statistics [13].

### Analysis

We used multilevel logistic regression analysis to explore the contribution of hospital and LHD of treatment, and SLA of residence to variation in nine low-value procedures used in adults in NSW (Table 1). The low-value procedures were selected from Choosing Wisely, RACP EVOLVE, and National Institute of Health and Care Excellence (NICE) ‘do not do’ recommendations as described previously [3, 9]. We analysed each of the procedures separately, as they cover a range of conditions, medical specialties, and populations, and there is no reason to expect effects would be the same across the low-value procedures.

**Table 1:** Definitions of the nine low value services

Service (recommendation source)	Narrower low value care definition	Broader low value care definition
Abdominal hysterectomy for benign disease (vs laparoscopic or vaginal) (Committee on Gynecologic Practice, 2009 [14])	Numerator: Denominator episodes involving hysterectomy with abdominal approach. Denominator: Episodes involving women aged 18 and older having hysterectomy, with no codes for caesarean, cancer, endometriosis or pelvic peritoneal adhesions recorded in the episode.	Numerator: Episodes involving hysterectomy with abdominal approach. Denominator: Episodes involving women aged 18 and older having hysterectomy, with no codes for caesarean or cancer recorded in the episode.
Arthroscopic lavage and debridement of knee for osteoarthritis or degenerative meniscal tears (CWUS, NICE)	Numerator: Denominator episodes involving knee arthroscopy. Denominator: Episodes involving people aged 55 or older with diagnosis of gonarthrosis or meniscal derangements and no diagnosis of ligament strain or damage or diagnosis of septic (pyogenic) arthritis recorded in the episode.	Numerator: Denominator episodes involving knee arthroscopy. Denominator: Episodes involving people aged 18 or older with diagnosis of gonarthrosis or meniscal derangements and no diagnosis of ligament strain or damage or septic (pyogenic) arthritis recorded in the episode.
Carotid endarterectomy for asymptomatic high-risk patients with limited life expectancy (CWA, EVOLVE, CWC, CWUS)	Numerator: Denominator episodes involving carotid endarterectomy. Denominator: Episodes involving people aged 18 or older with diagnosis of occlusion and stenosis of carotid artery or procedure of carotid endarterectomy with no excluding diagnosis in the episode, and ASA code 4–5 or (age ≥ 75 and ASA 3), and not emergency care or admitted through emergency department.	Numerator: Denominator episodes involving carotid endarterectomy. Denominator: Episodes involving people aged 18 or older with diagnosis of occlusion and stenosis of carotid artery or procedure of carotid endarterectomy with no excluding diagnosis in the episode, and ASA code 4–5 or age ≥ 75.
Colonoscopy for constipation in people < 50 years (CWC)	Numerator: Denominator episodes involving colonoscopy. Denominator: Episodes involving patients aged 18–49 with diagnosis of constipation, and no diagnoses of anaemia, weight loss, family or personal history of cancer of digestive system, or personal history of other diseases of the digestive system recorded in the previous 12 months.	Numerator: Denominator episodes involving colonoscopy. Denominator: Episodes involving patients aged 18–49 with diagnosis of constipation, and no diagnoses of anaemia, weight loss, family or personal history of cancer of digestive system, or personal history of other diseases of the digestive system recorded in the episode.
Endoscopic retrograde cholangiopancreatography (ERCP) without cholangitis (Tenner et al., 2013 [15]; Working Group IAP/APA Acute Pancreatitis Guidelines, 2013 [16]; Nesvaderani et al., 2015 [17])	Numerator: Denominator episodes involving ERCP. Denominator: Episodes involving patients aged 18 or older with diagnosis of calculus of bile duct or biliary acute pancreatitis, and no record of cholangitis and obstruction and not classed as emergency admission or admitted through the emergency department.	Numerator: Denominator episodes involving ERCP. Denominator: Episodes involving patients aged 18 or older with diagnosis of calculus of bile duct or biliary acute pancreatitis, and no record of cholangitis and obstruction.
Endoscopy for dyspepsia for people < 55 years (CWC)	Numerator: Denominator episodes involving endoscopy. Denominator: Episodes involving patients aged 18–54 with diagnosis of dyspepsia, and no excluding diagnoses recorded in the previous 12 months.	Numerator: Denominator episodes involving endoscopy. Denominator: Episodes involving patients aged 18–54 with diagnosis of dyspepsia, and no excluding diagnoses recorded in the episode.
Endovascular repair of infrarenal abdominal aortic aneurysm (CWC)	Numerator: Denominator episodes involving endovascular repair of aneurysm. Denominator: Episodes involving people aged 18 or older with diagnosis of abdominal aortic aneurysm, ASA score 4–5, or (age ≥ 75 and ASA 3), not emergency or admitted from emergency department.	Numerator: Denominator episodes involving endovascular repair of aneurysm. Denominator: Episodes involving people aged 18 or older with diagnosis of abdominal aortic aneurysm, ASA score 4–5, or age ≥ 75.
Sentinel lymph node biopsy for melanoma in situ or T1a melanoma (EVOLVE, CWUS)	Numerator: Denominator episodes involving sentinel lymph node biopsy. Denominator: Episodes involving patients aged 18 or older with diagnosis of melanoma in situ or melanoma (morphology code M872–M879 /0–2) and no other cancer code recorded in the episode.	Numerator: Denominator episodes involving sentinel lymph node biopsy. Denominator: Episodes involving patients aged 18 or older with diagnosis of melanoma in situ or melanoma (morphology code M872–M879 /0–3) and no other cancer code recorded in the episode.
Spinal fusion for low back pain (CWC)	Numerator: Denominator episodes involving spinal fusion. Denominator: Episodes involving patients aged 18 or older with diagnosis of low back pain with no mention of sciatica, spondylolisthesis or spinal deformity, or pain in legs recorded in the previous 12 months.	Numerator: Denominator episodes involving spinal fusion. Denominator: Episodes involving patients aged 18 or older with diagnosis of low back pain or spinal stenosis with no mention of sciatica, spondylolisthesis or spinal deformity, or pain in legs recorded in the episode.

Adapted from Badgery-Parker et al. (2018) Additional file 2: Table S1 [3].

For each of the procedures, denominator cohorts comprised all patients in NSW public hospitals that provide the procedure and for whom the procedure would be low value according to our definitions. The binary outcome variable was whether the procedure was provided. We developed both narrower and broader definitions of low-value care to account for uncertainty in whether episodes are truly low value [3]. Here, we present results for the narrower definitions; results for the broader definitions lead to similar conclusions.

**Fig. 1:**
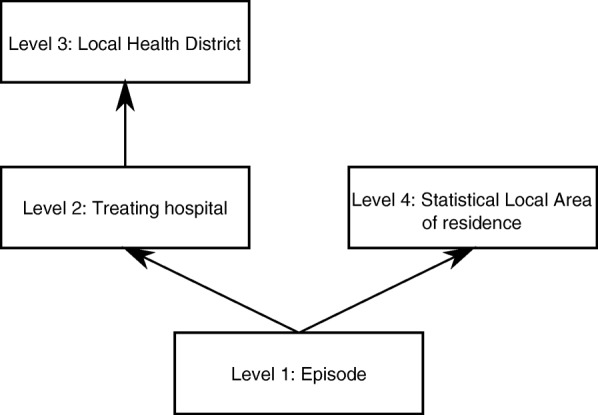
Multilevel structure used in the models. Most models included the four levels Episode, Treating hospital, Local Health District (LHD) of the treating hospital, and Statistical Local Area (SLA) of residence. Two procedures (spinal fusion, endovascular repair of abdominal aortic aneurysm) occurred at only one hospital per LHD, so the LHD level was excluded in modelling those procedures. Arrows show a nested relationship (each episode occurs at only one hospital; each hospital belongs to only one LHD; each patient lives in only one SLA). Cross-classification occurs between SLA and hospital and between SLA and LHD, meaning patients from the same SLA may be treated in different hospitals and LHDs, while the same hospital may treat patients from different SLAs

Our models involved four levels: episode, hospital, LHD, and SLA (Fig. 1; Additional file 1). Hospital and LHD have a hierarchical (nested) structure (each hospital belongs to only one LHD), and are cross-classified with SLA, meaning hospitals can treat patients from multiple SLAs, and patients from the same SLA can be treated in different hospitals and LHDs. Although it was possible for patients to have multiple episodes recorded in the data, less than 10% of records involved repeated admissions of the same patient. Furthermore, there was very little variation at the patient level and including this level led to problems with model convergence. Therefore, the models did not account for multiple episodes involving the same patient. Two procedures (spinal fusion and endovascular repair of abdominal aortic aneurysm [EVAR]) occurred at only one hospital in each LHD. In these cases, it is not reasonable to try to separate variation into the LHD or hospital contribution, so LHD was not included in the models for these two procedures.

For each procedure, we fit a series of models to examine the relative contributions of each level in explaining the rates of low-value care (Table 2) [18]. Model 1 included only variables at the episode level with no higher levels. This provided a baseline for assessing the contribution of hospital, LHD, and SLA. Models 2–4 separately added hospital, LHD, or SLA to Model 1, and Model 5 included all levels. These models were intended to explore the relative contributions of the different levels to explaining whether patients received low-value care. Finally, in Model 6 we added specific hospital-, LHD-, and SLA-level variables (Box 1) to Model 5, to assess whether any of these available variables explained the effect of hospital, LHD, or SLA. For the analysis, continuous variables were centred at the mean value for the cohort. There were no missing data for any of the variables used in the analysis.

**Table 2:** The six models fit for each procedure

	Model 1	Model 2	Model 3	Model 4	Model 5	Model 6
Model purpose	Base for comparing effects of adding levels	Effect of adding hospital to M1	Effect of adding LHD to M1	Effect of adding SLA to M1	1. Additional effect of including all 3 levels 2. Contribution of each level to variation in odds of receiving low value care	Effect of adding level-specific variables
Assess using	–	Increase in *c* statistic vs M1	Increase in *c* statistic vs M1	Increase in *c* statistic vs M1	1. Increase in *c* statistic vs M2, M3, and M4; 2. VPC, MOR	IOR80 OR
Variables included^b^						
Levels						
Hospital		X			X	X
LHD			X		X^a^	X^a^
SLA				X	X	X
Episode variables						
Age	X	X	X	X	X	X
Sex	X	X	X	X	X	X
Charlson	X	X	X	X	X	X
Private patient	X	X	X	X	X	X
Emergency care	X	X	X	X	X	X
Financial year	X	X	X	X	X	X
Hospital variables						
Hospital peer group						X
Procedures episodes as proportion total episodes						X
LHD variables						
Rural/metropolitan						X
SLA variables						
IRSAD quintile						X
Remoteness category						X
Population prevalence of low-value indication						X

^a^LHD was not included in Models 5 and 6 for spinal fusion and EVAR because each LHD had only one hospital that performed the procedure. ^b^ Additional file 1 contains more details about the variables

*IRSAD* Index of Relative Socioeconomic Advantage and Disadvantage, *LHD* Local Health District, *MOR* median odds ratio, *SLA* Statistical Local Area, *VPC* variance partition coefficient

We first compared Models 2–4 with Model 1 using the *c* statistic (equivalent to the area under the receiver–operator characteristic curve) [18, 19]. The *c* statistic is a measure of how well a model predicts which patients receive low-value care, and can range from 0.5 (indicating the model has no predictive skill) to 1 (indicating the model perfectly predicts which patients receive low-value care). For Models 2–4, the increase in *c* statistic from Model 1 is a measure of the additional information on whether an episode was low value provided by knowing the hospital, LHD, or SLA. The greater the change, the more important is knowing the hospital, LHD, or SLA for predicting if a patient did or did not receive the procedure. The increase in *c* statistic from Models 2–4 to Model 5 was also examined to assess the additional value of including all three levels.

We next assessed the relative contributions of the different levels in Model 5 using the variance partition coefficient (VPC) and the median odds ratio (MOR) [19]. The VPC is the percentage of residual variation attributable to a specific level. We calculated the VPC using the latent response approach, setting the level 1 variance to π^2^/3 [19, 20]. The higher the VPC, the greater the contribution of the level to the variation in the latent response at the individual level. The MOR converts the level variation to the odds ratio scale, allowing direct comparison with the odds ratios for other variables. The hospital MOR can be interpreted as the average increase in odds of receiving low-value care if a patient went to a different hospital (in the same LHD and while living in the same area) with higher low-value care rate. Similarly, the SLA MOR is the average increase in odds if a patient (attending the same hospital in the same LHD) lived in a different SLA with higher rate of low-value care. As it is not possible for a patient to attend the same hospital in a different LHD, we present the MOR for LHD + hospital, which indicates the average increase in odds if a patient went to a different hospital in a different LHD (while living in the same SLA).

Finally, for Model 6 we examined the effects of hospital, LHD, and SLA covariates. While the earlier models assessed if the different levels are important in explaining low-value care, Model 6 sought to identify variables through which the level effect occurs. We assessed the variables using the 80% interval odds ratio (IOR80) [19]. The IOR80 is required because, although the odds ratios are conditional on the hospital, LHD, and SLA in these multilevel models, the covariates are constant within any unit to which they apply (e.g. it is not possible to change from a peer group A1 hospital to a peer group B hospital while remaining at the same hospital). The IOR80 covers the middle 80% of odds ratios that would be observed when changing from one value of a level-specific covariate to another, accounting for the required change in the hospital, LHD, or SLA. When the IOR80 includes the value 1, the interval is “wide” and indicates that the covariate effect is minor compared with the residual variation at that model level [21].

### Software

We fit the models using RStan, accessed through the brms package in R 3.4.0, with weakly informative priors for the covariate parameters and brms default priors for the variance components.

### Ethics

This study was approved by the New South Wales Population and Health Services Research Ethics Committee.

## Results

Additional file 2 summarises the cohorts for the nine services. Sample sizes ranged from 1181 episodes for EVAR to 58,459 episodes for knee arthroscopy (Additional file 2: Table S1A).

Here we present results using our narrower definitions of low-value care. Additional file 3 includes results for the broader definitions, which lead to similar conclusions.

### Magnitude of level effects

For knee arthroscopy, ERCP, and spinal fusion, Model 1 (no multilevel structure) had a *c* statistic of ≥0.7, conventionally taken to indicate acceptable discrimination (Table 3) [22]. The other procedures all had *c* statistics between 0.6 and 0.7 for Model 1.

**Table 3:** *c* statistics for Models 1 to 5, and change between models

Low-value procedure	Model 1 *c*	Model 2 (hospital) *c* ∆M1 (95% CI)	Model 3 (LHD) *c* ∆M1 (95% CI)	Model 4 (SLA) *c* ∆M1 (95% CI)	Model 5 (all 3 levels)			
					*c*	∆M2	∆M3	∆M4
Sentinel lymph node biopsy for early melanoma	0.69	0.83	0.76	0.77	0.86	0.03	0.10	0.09
		0.14 (0.11–0.17)	0.07 (0.05–0.10)	0.08 (0.07–0.10)		(0.03–0.04)	(0.08–0.12)	(0.07–0.11)
Carotid endarterectomy in asymptomatic high risk patients	0.66	0.76	0.73	0.73	0.78	0.02	0.05	0.05
		0.10 (0.07–0.12)	0.07 (0.05–0.09)	0.07 (0.05–0.08)		(0.01 to 0.02)	(0.03–0.06)	(0.03–0.06)
EVAR in asymptomatic high risk patients	0.61	0.66	0.66	0.68	0.69	0.03	0.03	0.01
		0.06 (0.03–0.09)	0.05 (0.02–0.08)	0.08 (0.07–0.10)		(0.02 to 0.04)	(0.02 to 0.04)	(− 0.02 to 0.03)
Knee arthroscopy for osteoarthritis	0.75	0.83	0.78	0.78	0.83	0.00	0.06	0.05
		0.08 (0.07–0.08)	0.03 (0.02–0.03)	0.03 (0.03–0.03)		(0.00–0.01)	(0.05–0.06)	(0.05–0.05)
ERCP without cholangitis or obstruction	0.72	0.77	0.75	0.75	0.78	0.01	0.03	0.03
		0.05 (0.03–0.06)	0.02 (0.01–0.03)	0.021 (0.02–0.03)		(−0.01 to 0.01)	(0.02–0.04)	(0.02–0.05)
Abdominal hysterectomy	0.63	0.71	0.67	0.69	0.72	0.00	0.04	0.02
		0.09 (0.08–0.09)	0.04 (0.04–0.05)	0.06 (0.06–0.07)		(0.00–0.00)	(0.04–0.05)	(0.02–0.03)
Spinal fusion for low back pain	0.74	0.81	0.81	0.85	0.88	0.07	0.07	0.03
		0.07 (0.05–0.09)	0.06 (0.04–0.08)	0.10 (0.08–0.12)		(0.05–0.08)	(0.05–0.09)	(0.02–0.05)
Colonoscopy for constipation in people < 50 years	0.64	0.76	0.69	0.71	0.78	0.01	0.09	0.06
		0.13 (0.12–0.14)	0.05 (0.04–0.06)	0.07 (0.06–0.08)		(0.01–0.01)	(0.08–0.10)	(0.06–0.07)
Endoscopy for dyspepsia in people < 55	0.62	0.78	0.68	0.73	0.79	0.01	0.10	0.06
		0.16 (0.15–0.17)	0.06 (0.06–0.07)	0.10 (0.10–0.11)		(0.00–0.01)	(0.10–0.11)	(0.06–0.07)

For details of the models, see Table 2. *ERCP* endoscopic retrograde cholangiopancreatography, *EVAR* endovascular repair of abdominal aortic aneurysm

**Fig. 2:**
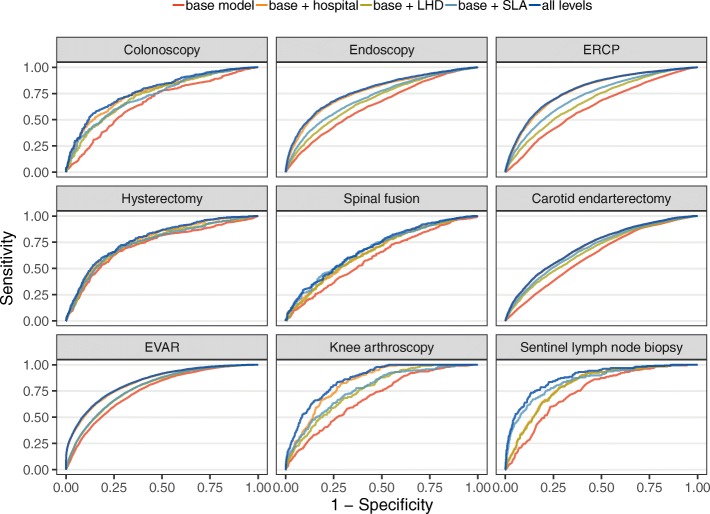
ROC curves for Models 1–5 for each service. ERCP, endoscopic retrograde cholangiopancreatography. EVAR, endovascular repair of abdominal aortic aneurysm. LHD, Local Health District. SLA, Statistical Local Area

Comparing Model 1 with Models 2–4 shows that adding information on the hospital provides a greater improvement to the model than adding LHD (without hospital) or SLA (Table 3; Fig. 2). That is, knowing the hospital improves prediction of whether a patient receives low-value care more than knowing the LHD or SLA. The increase in *c* statistic when adding hospital was greatest for endoscopy, colonoscopy, and sentinel lymph node biopsy. Models 3 and 4 show that LHD and SLA provide some information beyond the episode variables, but the increase in *c* statistic was smaller than for Model 2 (hospital). Model 5 shows that including all three levels provides no improvement in discrimination compared with just including hospital, except for spinal fusion, where including SLA provides a small additional increase in *c* compared with including only hospital.

Overall, these results indicate that each level provides information about whether patients will receive low-value care, but the hospital level is the most useful.

### Relative importance of the levels

**Fig. 3:**
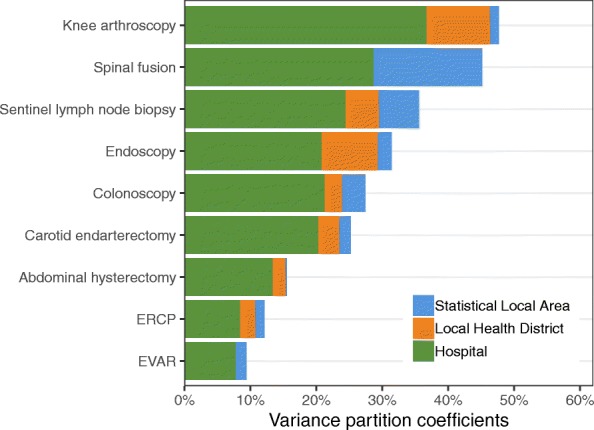
Proportion of residual variation at the hospital, Local Health District, and Statistical Local Area of residence levels. The total length of the bars shows the total proportion of variation explained by hospital, Local Health District, and Statistical Local Area. The remaining residual variation is attributable to unmeasured characteristics of the individual episode. LHD was not included in the models for EVAR and spinal fusion because of the small number of hospitals performing these procedures. ERCP, endoscopic retrograde cholangiopancreatography. EVAR, endovascular repair of abdominal aortic aneurysm

The VPCs from Model 5 for each procedure show that most of the residual variation after including the available episode-level variables is at the hospital level (Fig. 3; Table 4). EVAR had the lowest residual variation at the hospital level (7.8%; 95% CI, 2.9–15.8). The LHD accounted for more residual variation than the SLA for most procedures, although the VPCs for LHD and SLA were small and the uncertainty in the estimates was high (Table 4).

**Table 4:** Variance parameters for Model 5 for each of the nine procedures

Parameter	Sentinel lymph node biopsy for early melanoma	Carotid endarterectomy in asymptomatic high risk patients	EVAR in asymptomatic high risk patients	Knee arthroscopy for osteoarthritis	ERCP without cholangitis or obstruction	Abdominal hysterectomy	Spinal fusion for low back pain	Colonoscopy for constipation in people < 50	Endoscopy for dyspepsia for people < 55
Hospital level									
Variance	1.2 (0.6–2.5)	0.9 (0.4–1.7)	0.3 (0.1–0.7)	2.3 (1.6–3.3)	0.3 (0.1–0.7)	0.5 (0.3–0.8)	1.7 (0.6–4.3)	1.0 (0.7–1.4)	1.0 (0.7–1.3)
VPC	24.4 (15.6–32.1)	20.3 (11.9–28.4)	7.8 (2.9–15.8)	36.8 (31.9–39.0)	8.5 (3.9–14.7)	13.4 (9.6–17.3)	28.7 (12.8–47.0)	21.3 (16.4–25.6)	20.9 (17.5–23.2)
MOR	2.9 (2.1–4.5)	2.5 (1.9–3.5)	1.7 (1.3–2.2)	4.3 (3.3–5.7)	1.7 (1.4–2.2)	2.0 (1.8–2.3)	3.5 (2.0–7.3)	2.6 (2.2–3.1)	2.6 (2.3–3.0)
LHD level									
Variance	0.3 (0.0–1.2)	0.1 (0.0–0.8)		0.6 (0.1–1.8)	0.1 (0.0–0.5)	0.1 (0.0–0.3)		0.1 (0.0–0.5)	0.4 (0.1–1.0)
VPC	5.0 (0.1–16.1)	3.1 (0.0–13.4)		9.5 (1.5–20.8)	2.3 (0.0–9.9)	1.9 (0.0–7.0)		2.6 (0.0–9.2)	8.4 (1.8–17.5)
MOR	1.6 (1.0–2.9)	1.4 (1.0–2.4)		2.1 (1.3–3.6)	1.3 (1.0–1.9)	1.3 (1.0–1.7)		1.4 (1.0–2.0)	1.8 (1.4–2.6)
MOR (LHD + hospital)	3.2 (2.1–6.3)	2.6 (1.9–4.6)	1.7 (1.3–2.2)	5.1 (3.4–8.6)	1.8 (1.4–2.8)	2.1 (1.8–2.7)	3.5 (2.0–7.3)	2.7 (2.2–3.7)	3.1 (2.4–4.3)
SLA level									
Variance	0.3 (0.1–0.7)	0.1 (0.0–0.3)	0.1 (0.0–0.2)	0.1 (0.1–0.1)	0.1 (0.0–0.2)	0.0 (0.0–0.0)	1.0 (0.6–1.6)	0.2 (0.1–0.2)	0.1 (0.1–0.1)
VPC	6.1 (1.6–9.5)	1.8 (0.1–4.2)	1.6 (0.0–5.9)	1.4 (1.1–1.4)	1.4 (0.0–3.8)	0.2 (0.0–0.6)	16.4 (12.6–17.5)	3.6 (2.7–4.3)	2.2 (1.7–2.5)
MOR	1.7 (1.3–2.3)	1.3 (1.0–1.6)	1.3 (1.0–1.6)	1.3 (1.3–1.4)	1.2 (1.0–1.5)	1.1 (1.0–1.2)	2.6 (2.0–3.4)	1.5 (1.4–1.6)	1.4 (1.3–1.4)

For knee arthroscopy, the hospital, LHD, and SLA together accounted for 47.7% of the variation. At the other extreme, for EVAR these levels accounted for only 9.4% of the residual variation, and for ERCP, only 12.2% of the residual variation (Fig. 3).

**Fig. 4:**
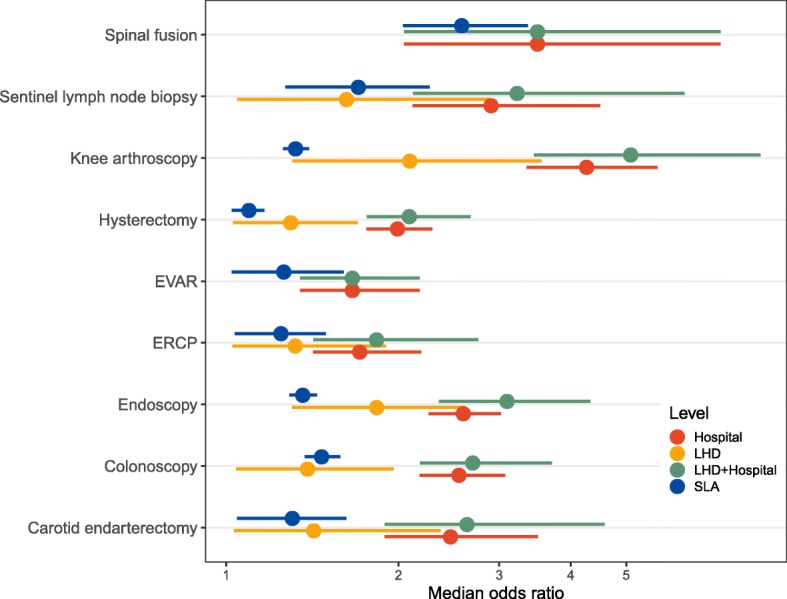
Median odds ratios (MORs). EVAR, endosvascular repair of aortic abdominal aneurysm. ERCP, endoscopic retrograde cholangiopancreatography. LHD, Local Health District. SLA, Statistical Local Area

For knee arthroscopy, the hospital MOR was 4.3 (95% CI, 3.3–5.7), indicating that the odds of a patient for whom the procedure is low value actually receiving the procedure would be 4.3 times higher on average if the patient attended a different hospital in the same LHD with a higher low-value rate (Table 4; Fig. 4). Hospital MORs for the other services were generally lower (most < 2.5). The MORs for SLA were between 1 and 2 for most procedures, but reached 2.6 (95% CI, 2.0–3.4) for spinal fusion.

### Level-specific variables

Patient age was associated with use of low-value care for all the procedures in Model 6, with the specific patterns depending on the service (Table 5). Higher Charlson comorbidity score was associated with lower odds of low-value care except in the case of hysterectomy, where they were associated with higher odds of abdominal rather than vaginal or laparoscopic hysterectomy. Private patients (in these public hospitals, as opposed to public patients in these public hospitals) had lower odds of low-value knee arthroscopy, colonoscopy, endoscopy, and spinal fusion, but higher odds of low-value ERCP, sentinel lymph node biopsy, and hysterectomy.

**Table 5:** Parameter estimates for Model 6 (full model) for the nine low-value procedures

Variable	Statistic	Sentinel lymph node biopsy	Carotid endarterectomy	EVAR	Knee arthroscopy	ERCP	Abdominal hysterectomy	Spinal fusion	Colonoscopy	Endoscopy
Episode level										
Age group*										
1	OR	18–39: 0.2	18–74: 0.8	18–74: 0.5	54–64: 2.1	18–39: 0.5	18–39: 0.7	18–49: 0.5	18–29: 0.6	18–24: 0.4
		(0.1–0.5)	(0.5–1.2)	(0.3–0.7)	(1.9–2.2)	(0.3–0.7)	(0.7–0.8)	(0.3–0.7)	(0.5–0.7)	(0.3–0.4)
2	OR	40–54: 0.9	75–79: 1	75–79: 1	65–79: 1	40–54: 0.6	40–49: 1	50–64: 1	30–34: 0.6	25–34: 0.5
		(0.6–1.3)				(0.4–0.8)			(0.5–0.7)	(0.5–0.5)
3	OR	55–69: 1	80–84: 0.8	80–84: 1.5	70–79: 0.5	55–69: 1	50–59: 0.8	65–79: 1.0	35–44: 0.9	35–44: 0.7
			(0.6–1.1)	(1.0–1.2)	(0.5–0.6)		(0.7–0.8)	(0.7–1.5)	(0.8–1.0)	(0.7–0.8)
4	OR	70–100: 0.8	85–99: 0.8	85–99: 0.9	80–110: 0.2	70–105: 1.9	60–95: 0.2	80–105: 0.1	45–49: 1	45–54: 1
		(0.6–1.1)	(0.5–1.2)	(0.6–1.4)	(0.1–0.2)	(1.4–2.6)	(0.2–0.3)	(0.1–0.3)		
Female	OR	0.6	1.0	0.7	0.9	1.0		1.0	1.4	1.0
		(0.4–0.8)	(0.7–1.3)	(0.5–1.0)	(0.8–1.0)	(0.8–1.2)		(0.8–1.5)	(1.3–1.6)	(0.9–1.0)
Charlson comorbidity index										
1	OR	1.2	0.5	0.9	0.7	0.7	1.1	0.7	0.4	0.5
		(0.7–2.0)	(0.3–0.7)	(0.6–1.3)	(0.6–0.7)	(0.5–1.1)	(0.9–1.3)	(0.4–1.1)	(0.3–0.5)	(0.5–0.6)
2–16	OR	0.8	0.3	0.8	0.2	1.0	1.8	0.2	0.3	0.2
		(0.4–1.8)	(0.2–0.4)	(0.6–1.1)	(0.2–0.3)	(0.7–1.4)	(1.3–2.4)	(0.1–0.3)	(0.2–0.4)	(0.2–0.3)
Private patient	OR	1.8	1.2	1.0	0.5	2.4	1.2	0.2	0.6	0.6
		(1.1–2.8)	(0.8–1.7)	(0.6–1.5)	(0.4–0.6)	(1.8–3.1)	(1.0–1.4)	(0.1–0.4)	(0.5–0.7)	(0.6–0.7)
Financial year	OR	1.1	1.1	1.1	0.9	1.0	0.9	1.0	1.0	1.0
		(1.0–1.2)	(1.0–1.2)	(1.0–1.1)	(0.9–0.9)	(0.9–1.0)	(0.9–1.0)	(0.9–1.1)	(0.9–1.0)	(1.0–1.0)
Hospital level										
Peer group										
B	OR	0.4	2.5	1.0	1.1	0.6	0.8	0.4	1.1	1.2
		(0.1–1.6)	(1.0–6.9)	(0.4–3.3)	(0.8–1.4)	(0.4–0.9)	(0.5–1.2)	(0.0–4.0)	(0.7–1.6)	(0.8–1.8)
	IOR80	0.1–2.7	0.6–11	0.5–2.2	0.1–13	0.4–0.9	0.2–2.6	0.1–3.5	0.4–3.0	0.4–4.0
C1	OR	0.4			2.2		1.2		1.2	0.7
		(0.1–1.9)			(0.9–5.1)		(0.6–2.1)		(0.7–2.0)	(0.4–1.1)
	IOR80	0.1–2.5			0.2–28		0.4–3.9		0.4–3.3	0.2–2.3
C2	OR	0.2			4.2		0.6		0.9	0.9
		(0.0–1.2)			(1.7–9.6)		(0.3–1.0)		(0.5–1.6)	(0.6–1.5)
	IOR80	0.0–1.1			0.3–52		0.2–1.8		0.3–2.6	0.3–3.0
D1a	OR				3.6				1.3	0.6
					(0.9–13)				(0.5–2.9)	(0.3–1.2)
	IOR80				0.3–45				0.5–3.5	0.2–2.0
D1b	OR								0.9	0.6
									(0.2–3.3)	(0.2–1.8)
	IOR80								0.3–2.5	0.2–1.8
Procedure as proportion total volume	OR	1.3	4.2	3.4	1.1	4.5	1.0	1.2	1.1	1.1
		(1.1–1.5)	(2.0–11.4)	(1.4–7.9)	(1.1–1.1)	(2.6–7.8)	(0.9–1.0)	(0.8–1.8)	(1.1–1.1)	(1.1–1.1)
	IOR80	0.2–8.5	1.0–19	1.6–7.2	0.1–14	2.9–7.1	0.3–3.3	0.1–11	0.4–3.0	0.3–3.6
*Variance*		1.1	0.7	0.2	2.0	0.1	0.4	1.5	0.3	0.4
		(0.4–2.4)	(0.3–1.4)	(0.0–0.5)	(1.4–2.8)	(0.0–0.2)	(0.3–0.7)	(0.5–3.8)	(0.2–0.5)	(0.3–0.6)
*VPC*		20	16	4.7	35	1.8	11	29	8.3	11
*MOR*		2.7 (1.8–4.3)	2.2 (1.6–3.1)	1.5 (1.2–1.9)	3.8 (3.0–5.0)	1.3 (1.1–1.6)	1.9 (1.7–2.2)	3.2 (2.0–6.5)	1.7 (1.5–2.0)	1.9 (1.7–2.1)
LHD level										
Rural	OR	1.5	1.0	1.1	2.1	1.0	1.0	0.2	1.4	1.3
		(0.4–7.3)	(0.3–4.3)	(0.4–2.8)	(0.7–6.2)	(0.5–1.9)	(0.6–1.9)	(0.0–2.0)	(0.9–2.3)	(0.7–2.2)
	IOR80	0.3–7.6	0.6–1.9		0.8–5.7	0.6–1.5	0.6–1.8		0.9–2.2	0.7–2.4
*Variance*		0.8	0.1		0.3	0.1	0.1		0.1	0.1
		(0.0–2.7)	(0.0–0.8)		(0.0–1.1)	(0.0–0.3)	(0.0–0.4)		(0.0–0.2)	(0.0–0.4)
*VPC*		14	2.8		5.2	1.9	2.8		1.6	2.9
*MOR*		2.3 (1.2–4.3)	1.4 (1.0–2.3)		1.7 (1.1–2.7)	1.3 (1.0–1.7)	1.4 (1.1–1.8)		1.3 (1.0–1.6)	1.4 (1.1–1.8)
SLA level										
IRSAD quintile										
2	OR	0.6	1.1	0.8	1.2	1.0	1.0	0.7	1.0	0.9
		(0.3–1.1)	(0.7–1.7)	(0.5–1.3)	(1.0–1.4)	(0.7–1.5)	(0.9–1.1)	(0.4–1.3)	(0.7–1.3)	(0.8–1.1)
	IOR80	0.2–1.8	0.6–2.0	0.5–1.2	0.7–2.0	0.6–1.6	0.8–1.2	0.2–2.1	0.5–2.0	0.5–1.8
3	OR	0.9	1.2	1.1	1.2	0.9	0.9	0.4	1.2	1.0
		(0.4–1.6)	(0.7–2.1)	(0.7–2.1)	(0.9–1.4)	(0.5–1.5)	(0.8–1.1)	(0.2–0.9)	(0.8–1.6)	(0.8–1.2)
	IOR80	0.3–2.5	0.6–2.2	0.7–1.8	0.7–2.0	0.5–1.5	0.8–1.1	0.1–1.2	0.6–2.4	0.5–1.8
4	OR	1.0	1.4	0.6	1.2	1.2	1.0	0.4	1.0	0.9
		(0.5–2.0)	(0.8–2.5)	(0.4–1.1)	(0.9–1.5)	(0.7–1.9)	(0.8–1.2)	(0.2–0.9)	(0.7–1.4)	(0.7–1.2)
	IOR80	0.3–2.8	0.7–2.6	0.4–1.0	0.7–2.0	0.7–1.9	0.8–1.2	0.1–1.3	0.5–2.0	0.5–1.7
5	OR	0.7	1.3	1.0	1.3	1.4	1.0	0.6	0.9	0.9
		(0.3–1.4)	(0.7–2.5)	(0.5–1.9)	(1.0–1.7)	(0.8–2.4)	(0.8–1.2)	(0.2–1.3)	(0.6–1.3)	(0.7–1.2)
	IOR80	0.2–1.9	0.7–2.4	0.6–1.6	0.8–2.2	0.8–2.2	0.8–1.2	0.2–1.8	0.4–1.9	0.5–1.6
Remoteness category										
Inner Regional	OR	2.5	0.6	0.9	0.7	1.5	1.1	3.8	1.0	1.3
		(1.4–4.7)	(0.4–1.0)	(0.5–1.4)	(0.6–0.9)	(0.9–2.5)	(0.9–1.3)	(2.0–6.7)	(0.8–1.4)	(1.0–1.6)
	IOR80	0.9–7.1	0.3–1.1	0.5–1.4	0.4–1.2	0.9–2.4	0.9–1.3	1.2–12	0.5–2.2	0.7–2.3
Outer Regional	OR	2.9	0.7	1.8	0.7	1.9	1.2	2.9	1.6	1.7
		(1.1–6.9)	(0.4–1.4)	(0.8–4.4)	(0.5–0.9)	(1.0–4.2)	(1.0–1.6)	(0.9–7.5)	(1.1–2.4)	(1.2–2.4)
	IOR80	1.0–8.4	0.4–1.3	1.1–2.9	0.4–1.2	1.2–3.2	1.0–1.5	0.9–9.1	0.8–3.3	0.9–3.1
Remote/Very remote	OR				0.6		1.0	3.3	0.9	1.6
					(0.3–1.3)		(0.6–1.6)	(0.2–30)	(0.3–2.4)	(0.8–3.0)
	IOR80				0.4–1.1		0.8–1.2	1.1–10.5	0.4–1.8	0.8–2.9
Population prevalence of indication	OR	1.1	1.3	0.9	1.0	1.1		0.7	0.9	0.9
		(0.3–4.4)	(0.5–3.0)	(0.4–1.9)	(0.9–1.1)	(0.9–1.3)		(0.5–1.1)	(0.9–1.0)	(0.8–1.0)
	IOR80	0.4–3.3	0.7–2.4	0.5–1.4	0.6–1.7	0.7–1.7		0.2–2.3	0.4–1.9	0.5–1.7
*Variance*		0.3	0.1	0.1	0.1	0.1	0.0	0.4	0.2	0.1
		(0.1–0.8)	(0.0–0.3)	(0.0–0.3)	(0.1–0.1)	(0.0–0.2)	(0.0–0.0)	(0.1–0.8)	(0.1–0.2)	(0.1–0.2)
*VPC*		6.2	2.8	2.0	1.5	2.1	0.3	7.7	4.3	2.9
*MOR*		1.7 (1.3–2.3)	1.4 (1.1–1.7)	1.3 (1.0–1.6)	1.3 (1.3–1.4)	1.3 (1.0–1.6)	1.1 (1.0–1.2)	1.8 (1.4–2.4)	1.5 (1.4–1.6)	1.4 (1.3–1.5)

*Age groups differ between services and are set at approximate quartiles for the cohort for each service. Individual age groups are specified before the parameter estimates. Reference categories were selected according to what seemed most appropriate for each procedure

*ERCP* endoscopic retrograde cholangiopancreatography, *EVAR* endovascular repair of abdominal aortic aneurysm, *IOR80* 80% interval odds ratio, *IRSAD* Index of Relative Socioeconomic Advantage and Disadvantage, *LHD* Local Health District, *MOR* median odds ratio, *OR* odds ratio, *SLA* Statistical Local Area, *VPC* variance partition coefficient

Variables at the hospital, LHD, and SLA levels included in Model 6 generally showed no significant effect (Table 5). The proportion of total episodes at a hospital that involved the relevant procedure had ORs > 1 for all the services, but this was only supported by the IOR80 for ERCP (IOR80, 2.9–7.1) and EVAR (IOR80, 1.6–7.2). For both these procedures, hospitals that do more of the procedure are more likely to perform the service inappropriately. Osteoarthritis patients from regional areas were less likely to have knee arthroscopy than those from Major Cities, but the IOR80s indicated this effect was not consistent across SLAs within the remoteness categories.

## Discussion

This multilevel analysis allowed us to partition the residual variation in low-value care between hospital, LHD, and SLA of residence. For all nine services we examined, hospital accounted for substantially more variation than LHD or SLA. Where a patient is treated appears more important than where they live in explaining their odds of receiving low-value care. The LHD to which a hospital belongs also showed little influence. These results have implications for future research and initiatives to reduce low-value care.

Geographic variation analysis is a common approach to investigating clinical variation and inappropriate care [1]. Projects such as the *Australian Atlas of Healthcare Variation* highlight areas where service utilisation is particularly high or low [4, 23]. This provides valuable insights into patterns of care across regions. However, our results suggest that this area-based approach will not be helpful for studying low-value care. In assessments of clinical variation for low-value care, area may be just a proxy for the hospitals where the residents are treated. Patients living in the same area may be more likely to attend the same few specialists, resulting in admission to the same few hospitals. Research effort to explain and reduce low-value care should focus more on factors within the hospital, rather than the areas where patients live.

Variation at the LHD level was also low compared with the hospital level. This could be interpreted as indicating LHDs have little influence at the level of clinical decision-making that governs low-value care. The main levers available to LHDs are funding allocation and development of policy and procedures for hospitals in the district. Unless a procedure is completely defunded or banned by policy, clinicians still have the authority to decide whether to provide it. Less restrictive policies and procedures aimed at reducing low-value care may be interpreted and implemented differently at different hospitals, diluting their effect.

Alternatively, lack of variation at the LHD level could indicate no LHD-wide initiatives have been tried. For knee arthroscopy, LHD-level variation was higher, and this procedure is one for which an LHD-based initiative has been documented [24]. A simple clinical governance process implemented at four hospitals in one LHD was associated with greater decrease in knee arthroscopy in that LHD compared with others. Thus, LHD-based initiatives can work, at least in some cases. However, it is important to note that actual implementation of the policy was at the hospital level, and another hospital in the LHD refused to participate, reportedly because staff at that hospital believed the procedure to be effective [24].

The hospital-, LHD-, and SLA-level variables we examined mostly showed little association with whether a patient receives low-value care. This implies the hospital effect occurs through other unmeasured or unobservable variables at the hospital level. These may include difficult-to-measure concepts such as ‘hospital culture’, local policies, and access to technological infrastructure. We included one variable, the procedure volume as proportion of total hospital volume, on the basis that hospitals that do more of the procedure overall may be more likely to do more low-value care for that procedure. The ORs were > 1 for all the procedures, tending to support this hypothesis. However, the IOR80s usually included 1, indicating that variation between hospitals overwhelms this effect — individual hospitals with a higher proportion of the procedure may still have lower low-value rates than other hospitals with a lower proportion. The exceptions were EVAR and ERCP — hospitals that do more of these procedures (in relation to their total volume) are substantially more likely to perform these procedures when they are not appropriate according to our definitions of low-value care.

One notable unmeasured factor is the treating clinician. This is essentially another, unobserved, level in our multilevel structure, with different clinicians treating patients within the same hospital, and the same clinicians treating patients at different hospitals. This is likely to explain a substantial proportion of the variation, as it is the clinician who ultimately decides what treatment to provide, although this decision will be influenced by hospital policies and colleagues’ opinions and practices. Our ethics approval did not allow access to clinician identifiers for this study, so we could not explore the effect of clinicians. This is likely to be a productive area for future research.

### Limitations

Our results must be influenced by using only hospital data. Although our analysis showed hospital has the greatest impact, SLA may have a greater impact overall when the possibility that some patients with the relevant conditions are managed in the community instead of hospital is considered. Patients who receive appropriate non-hospital care will not appear in our hospitalised cohorts. This is unlikely to make much practical difference — initiatives to reduce low-value care can still be targeted at the hospital level, and should ultimately increase appropriate care in the appropriate setting.

Another limitation is that patients are not necessarily in hospital for treatment of the indication that makes a procedure low value. For example, many older patients may have knee osteoarthritis recorded but be in hospital for treatment of another condition. We would still count them in our knee arthroscopy cohort as patients for whom arthroscopy is low value, even though there may never have been any possibility they would be offered this procedure because the treatment focus is on another condition. Thus, SLAs or hospitals with more patients with osteoarthritis would show lower rates of low-value knee arthroscopy. Coding rules specify that only diagnoses relevant to management of the patient should be recorded, and this should mitigate this issue. However, if osteoarthritis limited mobility and increased risk of falls, it could be relevant to the management of any condition for which the patient was being treated.

We focused this analysis on the variation at the different model levels. Nevertheless, the episode-level variables often had strong associations with low-value care. These associations may indicate limitations of our measures or areas where there is genuine clinical uncertainty. For example, colonoscopy for constipation in people younger than 50 years showed a steady increase as age increased towards 50. This recommendation is from Canada, and while it does not conflict with any Australian guidelines it simply may not be a recognised recommendation so not routinely followed in Australia. The age trend may indicate that clinicians recognise the investigation is not appropriate in younger people, but have differing ideas on what an appropriate age limit should be.

## Conclusion

Low-value care shows substantial variation by hospital, and with little variation by LHD of the treating hospital or SLA of patient’s residence. Investigations into the drivers of low-value care and initiatives to reduce low-value care might best be targeted at the hospital level. For a proper assessment of clinical variation in low-value care, we need to measure factors at the hospital level, such as culture, local policies, and access to technological infrastructure. In particular, we should investigate the practices, knowledge, and attitudes of individual clinicians. Our systems do not currently allow this on a broad scale, but it is likely to be key to truly understanding the factors that drive low-value care. While the geographic approach of measuring procedures within areas has provided some insights, future studies should adopt a service-delivery perspective. Measurement at the service-provider level (rather than the geographic region) is more likely to enable action to reduce clinical variation and low-value care.

## Additional files

Additional file 1: Descriptions of the model levels and variables used at each level. (PDF 22 kb)

Additional file 2: Table S1: Summary of cohorts for the 9 low-value services. (XLSX 22 kb)

Additional file 3: Results for analyses using the broader definitions of low-value care. (PDF 472 kb)

## Abbreviations

ERCP: Endoscopic retrograde cholangiopancreatography
EVAR: Endovascular repair of abdominal aortic aneurysm
IOR80: 80% interval odds ratio
IRSAD: Index of Relative Socioeconomic Advantage and Disadvantage
LHD: Local Health District
MOR: Median odds ratio
NSW: New South Wales
SLA: Statistical Local Area
VPC: Variance partition coefficient
