# Supplementary material for: Exploring variation in low-value care: a multilevel modelling study
Source: BMC Health Serv Res. 2019 May 30;19:345. doi: 10.1186/s12913-019-4159-1 (PMC6543591; doi:10.1186/s12913-019-4159-1)
Supplement: Supplementary file 1 — Descriptions of the model levels and variables used at each level. (PDF 22 kb) [file 12913_2019_4159_MOESM1_ESM.pdf]

Additional file 1: Descriptions of the model levels and variables used at each level

Our models involved four levels: episode, hospital, Local Health District (LHD), and Statistical Local Area (SLA). Hospital and LHD have a hierarchical (nested) structure (each hospital belongs to only one LHD), and are cross-classified with SLA, meaning hospitals can treat patients from multiple SLAs, and patients from the same SLA can be treated in different hospitals and LHDs.

Level 1: Episode

The episode is the lowest level in our analysis. Variables at this level are characteristics of the patient being treated and administrative details of the episode.

- Age (classified in approximate quartiles for each procedure)
- Sex
- Charlson comorbidity index (classified as 0, 1, ≥ 2)
- Private or public patient
- Financial year (episode end date)

Level 2: Treating hospital

Low-value care at the hospital level will reflect the practice of the specific clinicians working at that hospital. (Individual clinician data is not available for this study.)

Hospitals may also have individual policies around specific services, which could lead to differences in low-value care.

Some variation between hospitals is likely to result from differences in patient population that are not captured in the episode-level variables.

- Hospital peer group. This is a classification of hospitals that incorporates volume of episodes, types of services provided, and rural or non-rural location. A: Principal referral (>35,000 episodes per year, offering highly specialised services). B: Major hospitals (10,000–35,000 episodes per year). C1: District group 1 (4000–10,000 episodes per year). C2: District group 2 (2000–4000 episodes per year). D1a: Community acute with surgery (200–2000 episodes per year, at least 2% of episodes being surgical). D1b: Community acute without surgery (>200 episodes per year with <2% surgical; or <200 episodes per year).

- Proportion of total episodes at the hospital in the year that involve the specific procedure. This was initially calculated as procedure episodes per 1000 total episodes. For some procedures it was rescaled (multiplied or divided by 10) to produce parameter estimates of the same order of magnitude as the other parameters, to improve model stability.

Level 3: Local Health District

In New South Wales, 15 LHDs manage the hospitals and population health initiatives of a geographic area. The LHDs negotiate funding and performance agreements with the

Ministry of Health and can develop policies that apply to all hospitals within the LHD. Differences in performance agreements or LHD policies could lead to differences in low-value care between LHDs. Furthermore, if low-value care is driven by clinicians and they work in multiple hospitals within an LHD, this would decrease the impact of hospital and increase the impact of LHD.

- Rural or metropolitan

Level 4: Statistical Local Area of residence

Different areas of residence could have differences in low-value care due to differences in population that are not captured by the episode-level variables. In NSW, there are 199 SLAs, with estimated residential populations ranging from about 400 to about 155,000 (median, approximately 23,000).

In addition, although people can attend any general practice, a large proportion of people in an area are likely to attend one of a few local general practitioners (GPs). Thus, low-value care can be affected by the decisions of the GPs and the specialists to whom they refer patients.

- Index of Relative Socioeconomic Advantage and Disadvantage (IRSAD) quintile of the SLA. The IRSAD summarises the economic and social conditions of people residing in an area on a relative scale from most disadvantaged to most advantaged. It is constructed from 25 variables from Australian census data, such as the proportion of people with high income, the proportion with tertiary qualifications, the proportion unemployed, and the proportion of households with no car.
- Remoteness category of the SLA (if an SLA crossed multiple remoteness categories, the whole SLA was assigned to the predominant category). There are five remoteness categories based on a measure of relative access to services. The categories are Major Cities of Australia, Inner Regional Australia, Outer Regional Australia, Remote Australia, and Very Remote Australia. Because of small populations, Remote and Very Remote were combined for analysis, and if necessary further combined with Outer Regional.
- Prevalence of patients hospitalised with the indication for which the procedure is low value. This is the number of episodes involving the low-value indication per 1000 residents of the SLA.
