# Supplementary material for: Exploring variation in low-value care: a multilevel modelling study
Source: BMC Health Serv Res. 2019 May 30;19:345. doi: 10.1186/s12913-019-4159-1 (PMC6543591; doi:10.1186/s12913-019-4159-1)
Supplement: Supplementary file 3 — Results for analyses using the broader definitions of low-value care. (PDF 472 kb) [file 12913_2019_4159_MOESM3_ESM.pdf]

Additional file 3: Results for the broader definitions of low-value care

Supplementary Table 2: *c* statistics for Models 1 to 5, and change between models, broader definitions of low-value care

Low-value procedure	Model 1	Model 2 (hospital)	Model 3 (LHD)	Model 4 (SLA)	Model 5 (all 3 levels)			
	c	*c*<br>∆M1 (95% CI)	*c*<br>∆M1 (95% CI)	*c*<br>∆M1 (95% CI)	*c*	∆M2	∆M3	∆M4
Sentinel lymph node biopsy for early melanoma	0.64	0.79<br>0.14 (0.14–0.15)	0.73<br>0.09 (0.09–0.10)	0.70<br>0.05 (0.04–0.06)	0.81	0.01<br>(0.01–0.02)	0.06<br>(0.06–0.07)	0.11<br>(0.10–0.12)
Carotid endarterectomy in asymptomatic high risk patients	0.69	0.76<br>0.07 (0.06–0.07)	0.75<br>0.06 (0.05–0.06)	0.75<br>0.04 (0.03–0.05)	0.77	0.01<br>(0.00–0.02)	0.02<br>(0.01–0.03)	0.03<br>(0.02–0.04)
EVAR in asymptomatic high risk patients	0.65	0.68<br>0.03 (0.03–0.04)	0.67<br>0.03 (0.02–0.03)	0.70<br>0.04 (0.03–0.05)	0.72	0.02<br>(0.02–0.03)	0.03<br>(0.02–0.04)	0.02<br>(0.01–0.03)
Knee arthroscopy for osteoarthritis	0.80	0.86<br>0.05 (0.05–0.05)	0.82<br>0.02 (0.02–0.02)	0.82<br>0.02 (0.02–0.02)	0.86	0.00<br>(0.00–0.00)	0.04<br>(0.04–0.04)	0.04<br>(0.04–0.04)
ERCP without cholangitis or obstruction	0.65	0.67<br>0.02 (0.01–0.02)	0.67<br>0.01 (0.00–0.01)	0.68<br>0.02 (0.01–0.03)	0.70	0.01<br>(0.00–0.02)	0.02<br>(0.01–0.03)	0.01<br>(0.00–0.03)
Abdominal hysterectomy	0.61	0.70<br>0.09 (0.09–0.10)	0.66<br>0.06 (0.05–0.06)	0.68<br>0.07 (0.06–0.07)	0.71	0.00<br>(0.00–0.00)	0.04<br>(0.04–0.04)	0.03<br>(0.03–0.03)
Spinal fusion for low back pain	0.69	0.72<br>0.03 (0.03–0.03)	0.72<br>0.03 (0.03–0.03)	0.76<br>0.06 (0.06–0.07)	0.78	0.04<br>(0.04–0.05)	0.05<br>(0.04–0.05)	0.01<br>(0.01–0.02)
Colonoscopy for constipation in people < 50 years	0.63	0.76<br>0.13 (0.12–0.13)	0.68<br>0.05 (0.05–0.05)	0.70<br>0.07 (0.06–0.07)	0.77	0.01<br>(0.01–0.01)	0.08<br>(0.08–0.09)	0.07<br>(0.06–0.07)
Endoscopy for dyspepsia in people < 55	0.63	0.78<br>0.15 (0.15–0.15)	0.69<br>0.06 (0.06–0.06)	0.73<br>0.09 (0.09–0.10)	0.79	0.00<br>(0.00–0.01)	0.10<br>(0.10–0.10)	0.06<br>(0.06–0.06)

For details of the models, see Table 2. ERCP, endoscopic retrograde cholangiopancreatography. EVAR, endovascular repair of abdominal aortic aneurysm.

Supplementary Table 3: Variance parameters for Model 5 for each of the nine procedures, broader definitions of low-value care

Parameter	Sentinel lymph node biopsy for early melanoma	Carotid endarterectomy in asymptomatic high risk patients	EVAR in asymptomatic high risk patients	Knee arthroscopy for osteoarthritis	ERCP without cholangitis or obstruction	Abdominal hysterectomy	Spinal fusion for low back pain	Colonoscopy for constipation in people < 50	Endoscopy for dyspepsia for people < 55
**Hospital level**									
Variance	1.7 (1.0–3.0)	0.5 (0.2–1.1)	0.5 (0.2–1.1)	2.2 (1.6–3.0)	0.1 (0.1–0.3)	0.5 (0.3–0.7)	0.7 (0.3–1.6)	0.9 (0.6–1.3)	1.0 (0.7–1.4)
VPC	31.2 (22.1–37.1)	11.8 (4.9–19.0)	13.0 (6.1–24.1)	36.4 (31.6–38.4)	3.6 (1.8–6.5)	12.5 (8.8–16.6)	16.5 (8.0–28.4)	20.4 (15.9–24.1)	21.0 (17.8–23.2)
MOR	3.5 (2.6–5.2)	1.9 (1.5–2.7)	2.0 (1.6–2.8)	4.1 (3.3–5.2)	1.4 (1.3–1.6)	1.9 (1.7–2.3)	2.3 (1.7–3.3)	2.5 (2.2–2.9)	2.6 (2.3–3.1)
**LHD level**									
Variance	0.3 (0.0–1.5)	0.3 (0.0–1.0)		0.4 (0.0–1.4)	0.0 (0.0–0.1)	0.1 (0.0–0.4)		0.1 (0.0–0.5)	0.4 (0.1–1.1)
VPC	5.6 (0.1–18.4)	6.6 (0.2–18.5)		7.2 (0.7–18.1)	0.7 (0.0–3.5)	3.0 (0.3–8.5)		2.5 (0.0–9.7)	8.1 (1.3–18.6)
MOR	1.7 (1.1–3.2)	1.6 (1.1–2.6)		1.9 (1.2–3.1)	1.2 (1.0–1.4)	1.4 (1.1–1.8)		1.4 (1.0–2.0)	1.8 (1.2–2.7)
MOR (LHD+hospital)	3.9 (2.6–7.5)	2.3 (1.5–4.0)	2.0 (1.6–2.8)	4.7 (3.3–7.4)	1.5 (1.3–1.8)	2.1 (1.7–2.7)	2.3 (1.7–3.3)	2.6 (2.2–3.6)	3.1 (2.3–4.5)
**SLA level**									
Variance	0.2 (0.1–0.3)	0.1 (0.0–0.2)	0.2 (0.1–0.3)	0.1 (0.1–0.1)	0.1 (0.0–0.2)	0.0 (0.0–0.0)	0.5 (0.4–0.6)	0.2 (0.1–0.2)	0.1 (0.1–0.2)
VPC	3.4 (2.5–3.6)	1.7 (0.3–3.3)	4.8 (3.1–6.5)	1.4 (1.2–1.4)	3.0 (1.4–5.0)	0.5 (0.2–0.7)	10.7 (9.1–11.6)	3.5 (2.8–4.1)	2.3 (1.–2.5)
MOR	1.5 (1.4–1.7)	1.3 (1.1–1.5)	1.5 (1.4–1.7)	1.3 (1.3–1.4)	1.4 (1.2–1.5)	1.1 (1.1–1.2)	1.9 (1.8–2.1)	1.5 (1.4–1.6)	1.4 (1.3–1.4)

Supplementary Table 4: Parameter estimates for Model 6 (full model) for the 9 low-value procedures, broader definitions of low-value care

Variable	Statistic	Sentinel lymph node biopsy	Carotid endarterectomy	EVAR	Knee arthroscopy	ERCP	Abdominal hysterectomy	Spinal fusion	Colonoscopy	Endoscopy
**Episode level**										
Age group*										
1	OR	18–39: 0.6 (0.4–0.7)	18–74: 1.2 (0.8–1.7)	18–74: 0.9 (0.7–1.2)	18–64: 4.0 (3.8–4.3)	18–39: 0.5 (0.4–0.6)	18–39: 0.7 (0.7–0.8)	18–49: 0.5 (0.4–0.5)	18–29: 0.7 (0.6–0.8)	18–24: 0.4 (0.4–0.4)
2	OR	40–54: 0.9 (0.8–1.1)	75–79: 1	75–79: 1	65–79: 1	40–54: 0.6 (0.5–0.7)	40–49: 1	50–64: 1	30–34: 0.7 (0.6–0.8)	25–34: 0.5 (0.5–0.5)
3	OR	55–69: 1	80–84: 0.9 (0.8–1.2)	80–84: 0.9 (0.8–1.0)	70–79: 0.5 (0.5–0.6)	55–69: 1	50–59: 0.9 (0.8–0.9)	65–79: 1.1 (1.0–1.2)	35–44: 1.0 (0.9–1.1)	35–44: 0.8 (0.7–0.8)
4	OR	70–100: 0.5 (0.4–0.6)	85–99: 0.5 (0.4–0.7)	85–105: 0.4 (0.4–0.5)	80–110: 0.2 (0.1–0.2)	70–105: 1.6 (1.3–1.9)	60–95: 0.3 (0.3–0.3)	80–105: 0.3 (0.2–0.4)	45–49: 1	45–54: 1
Female	OR	0.6 (0.6–0.7)	0.7 (0.6–0.9)	0.6 (0.5–0.7)	0.8 (0.7–0.8)	0.9 (0.8–1.0)		0.9 (0.8–1.0)	1.4 (1.3–1.5)	1.0 (0.9–1.0)
Charlson comorbidity index										
1	OR	0.9 (0.7–1.2)	0.4 (0.3–0.6)	0.7 (0.6–0.9)	0.5 (0.5–0.6)	0.7 (0.6–1.0)	1.1 (1.0–1.3)	0.8 (0.7–0.9)	0.5 (0.4–0.6)	0.5 (0.5–0.6)
2–16	OR	0.5 (0.3–0.8)	0.3 (0.2–0.3)	0.6 (0.5–0.7)	0.2 (0.2–0.2)	0.9 (0.7–1.1)	1.8 (1.4–2.4)	0.4 (0.4–0.5)	0.3 (0.3–0.4)	0.3 (0.2–0.3)
Private patient	OR	0.8 (0.6–1.0)	0.7 (0.6–0.9)	0.9 (0.7–1.0)	0.5 (0.4–0.6)	1.5 (1.3–1.8)	1.3 (1.1–1.4)	0.5 (0.4–0.6)	0.6 (0.5–0.7)	0.6 (0.6–0.7)
Financial year	OR	1.2 (1.1–1.2)	1.1 (1.0–1.1)	1.1 (1.0–1.1)	0.9 (0.9–0.9)	1.0 (0.9–1.0)	0.9 (0.9–0.9)	1.1 (1.0–1.1)	1.0 (0.9–1.0)	1.0 (1.0–1.1)
**Hospital level**										
Peer group										
B	OR	0.3 (0.1–1.0)	1.0 (0.5–2.0)	0.5 (0.3–0.8)	1.1 (0.8–1.4)	0.8 (0.6–1.0)	0.8 (0.5–1.1)	0.4 (0.2–0.8)	1.2 (0.8–1.7)	1.3 (0.9–1.8)
	IOR80	0.0–2.3	0.4–3.0	0.3–0.8	0.1–11	0.6–1.0	0.2–2.5	0.2–0.7	0.5–3.1	0.4–4.2
C1	OR	0.3 (0.1–1.0)			2.6 (1.2–5.5)		1.2 (0.7–2.0)		1.3 (0.8–2.1)	0.7 (0.4–1.1)
	IOR80	0.0–2.1			0.3–26		0.4–3.8		0.5–3.4	0.2–2.3

C2	OR	0.1 (0.0–0.6)			3.7 (1.7–7.8)		0.6 (0.4–1.1)		1.2 (0.7–2.0)	0.9 (0.6–1.6)
	IOR80	0.0–0.9			0.4–37		0.2–2.0		0.4–3.2	0.3–3.1
D1a	OR				5.1 (1.8–16)				1.3 (0.6–2.9)	0.7 (0.3–1.3)
	IOR80				0.5–52				0.5–3.4	0.2–2.2
D1b	OR								0.9 (0.3–3.1)	0.6 (0.2–2.0)
	IOR80								0.4–2.4	0.2–2.0
Procedure as proportion total volume	OR	1.3 (1.2–1.4)	5.9 (3.3–11)	4.0 (2.7–5.8)	1.1 (1.1–1.1)	4.3 (3.1–6.1)	1.0 (1.0–1.0)	1.5 (1.3–1.7)	1.1 (1.1–1.1)	1.1 (1.1–1.1)
	IOR80	0.2–9.4	2.0–17	2.3–6.7	0.1–11	3.4–5.5	0.3–3.2	0.8–2.6	0.4–2.8	0.3–3.6
Variance		1.2 (0.5–2.7)	0.4 (0.1–0.8)	0.1 (0.0–0.3)	1.6 (1.1–2.5)	0.0 (0.0–0.1)	0.4 (0.3–0.7)	0.1 (0.0–0.3)	0.3 (0.2–0.5)	0.4 (0.3–0.7)
VPC		21	9.4	2.4	31	0.5	11	2.6	7.4	11
MOR		2.9 (2.0–4.8)	1.8 (1.4–2.3)	1.3 (1.2–1.7)	3.4 (2.7–4.5)	1.1 (1.0–1.3)	1.9 (1.6–2.2)	1.3 (1.2–1.7)	1.7 (1.5–1.9)	1.9 (1.7–2.2)
**LHD level**										
Rural	OR	3.2 (0.8–15)	1.1 (0.4–2.6)	0.8 (0.5–1.5)	2.0 (0.7–5.0)	0.9 (0.6–1.3)	1.1 (0.6–2.0)	0.7 (0.4–1.1)	1.3 (0.9–2.0)	1.3 (0.7–2.3)
	IOR80	0.5–21	0.6–1.7		0.8–4.7	0.8–1.1	0.6–2.4		0.9–1.9	0.7–2.5
Variance		1.1 (0.1–4.5)	0.1 (0.0–0.6)		0.2 (0.0–1.3)	0.0 (0.0–0.1)	0.2 (0.0–0.5)		0.0 (0.0–0.2)	0.1 (0.0–0.5)
VPC		19	1.9		4.5	0.3	4.1		1.0	3.2
MOR		2.7 (1.3–7.6)	1.3 (1.0–2.1)		1.6 (1.0–2.9)	1.1 (1.0–1.3)	1.5 (1.1–2.0)		1.2 (1.0–1.6)	1.4 (1.1–1.9)
**SLA level**										
IRSAD quintile										
2	OR	0.9 (0.6–1.2)	1.0 (0.7–1.5)	0.9 (0.7–1.3)	1.2 (1.0–1.4)	1.0 (0.7–1.3)	1.0 (0.9–1.1)	1.1 (0.8–1.5)	1.1 (0.8–1.4)	1.0 (0.8–1.2)
	IOR80	0.4–1.9	0.6–1.8	0.5–1.8	0.7–1.9	0.5–1.7	0.8–1.3	0.5–2.6	0.5–2.2	0.5–1.8
3	OR	0.8	1.1	1.0	1.1	0.8	1.0	0.9	1.2	1.0

		(0.6–1.1)	(0.7–1.6)	(0.7–1.4)	(0.9–1.3)	(0.6–1.2)	(0.9–1.1)	(0.6–1.3)	(0.9–1.5)	(0.8–1.3)
	IOR80	0.4–1.7	0.6–1.9	0.5–2.0	0.7–1.8	0.5–1.5	0.8–1.3	0.4–2.1	0.6–2.4	0.6–1.9
4	OR	0.9	1.0	0.9	1.1	1.0	1.1	0.6	1.1	1.0
		(0.6–1.4)	(0.7–1.5)	(0.6–1.2)	(0.9–1.4)	(0.7–1.4)	(0.9–1.2)	(0.4–0.9)	(0.8–1.4)	(0.8–1.2)
	IOR80	0.4–2.0	0.6–1.7	0.4–1.6	0.7–1.9	0.5–1.7	0.8–1.4	0.3–1.5	0.5–2.2	0.5–1.8
5	OR	0.6	0.7	0.9	1.3	1.1	1.1	0.7	1.0	1.0
		(0.4–0.9)	(0.4–1.1)	(0.6–1.4)	(1.0–1.6)	(0.7–1.6)	(0.9–1.2)	(0.5–1.1)	(0.7–1.4)	(0.7–1.3)
	IOR80	0.3–1.2	0.4–1.2	0.5–1.7	0.8–2.1	0.6–1.9	0.8–1.4	0.3–1.8	0.5–2.1	0.5–1.8
Remoteness category										
Inner Regional	OR	1.5	0.9	1.5	0.7	1.4	1.2	2.1	1.0	1.3
		(1.1–2.0)	(0.6–1.3)	(1.1–2.0)	(0.6–0.8)	(0.9–2.0)	(1.0–1.4)	(1.6–2.7)	(0.8–1.3)	(1.0–1.6)
	IOR80	0.7–3.2	0.5–1.6	0.8–2.9	0.4–1.2	0.8–2.4	0.9–1.5	0.9–5.1	0.5–2.1	0.7–2.3
Outer Regional	OR	1.3	1.1	2.7	0.6	1.6	1.1	2.7	1.5	1.6
		(0.8–2.2)	(0.6–1.8)	(1.7–4.5)	(0.5–0.8)	(0.9–3.0)	(0.9–1.4)	(1.9–4.0)	(1.1–2.2)	(1.2–2.2)
	IOR80	0.6–2.9	0.6–1.9	1.4–5.3	0.4–1.0	0.9–2.9	0.9–1.4	1.1–6.6	0.7–3.2	0.9–3.1
Remote/Very remote	OR				0.8		1.1	3.1	0.7	1.4
					(0.5–1.4)		(0.7–1.7)	(0.9–9.1)	(0.3–1.6)	(0.8–2.7)
	IOR80				0.5–1.4		0.9–1.4	1.3–7.5	0.3–1.4	0.8–2.6
Population prevalence of indication	OR	0.5<br>(0.3–0.9)	1.0<br>(0.5–2.0)	0.9<br>(0.6–1.4)	1.0<br>(0.9–1.1)	1.1<br>(0.9–1.2)		0.8<br>(0.7–0.9)	1.0<br>(0.9–1.0)	1.0<br>(0.9–1.0)
	IOR80	0.2–1.1	0.6–1.8	0.5–1.7	0.6–1.7	0.6–1.9		0.3–2.0	0.5–2.0	0.5–1.8
Variance		0.2	0.1	0.1	0.1	0.1	0.0	0.2	0.2	0.1
		(0.1–0.3)	(0.0–0.3)	(0.1–0.3)	(0.1–0.1)	(0.0–0.2)	(0.0–0.0)	(0.1–0.4)	(0.1–0.2)	(0.1–0.2)
VPC		3.2	2.5	3.9	1.5	3.0	0.5	6.5	4.3	2.9
MOR		1.5 (1.3–1.7)	1.3 (1.1–1.6)	1.4 (1.3–1.6)	1.3 (1.2–1.4)	1.4 (1.2–1.5)	1.1 (1.1–1.2)	1.6 (1.5–1.8)	1.5 (1.4–1.6)	1.4 (1.3–1.5)

* Age groups differ between services and are set at approximate quartiles for the cohort for each service. Individual age groups are specified before the parameter estimates. Reference categories were selected according to what seemed most appropriate for each procedure. ERCP, endoscopic retrograde cholangiopancreatography. EVAR, endovascular repair of abdominal aortic aneurysm. IOR80, 80% interval odds ratio. IRSAD, Index of Relative Socioeconomic Advantage and Disadvantage. LHD, Local Health District. MOR, median odds ratio. OR, odds ratio. SLA, Statistical Local Area. VPC, variance partition coefficient.

Supplementary Figure 1: ROC curves for Models 1–5 for each service, broader definitions of low-value care

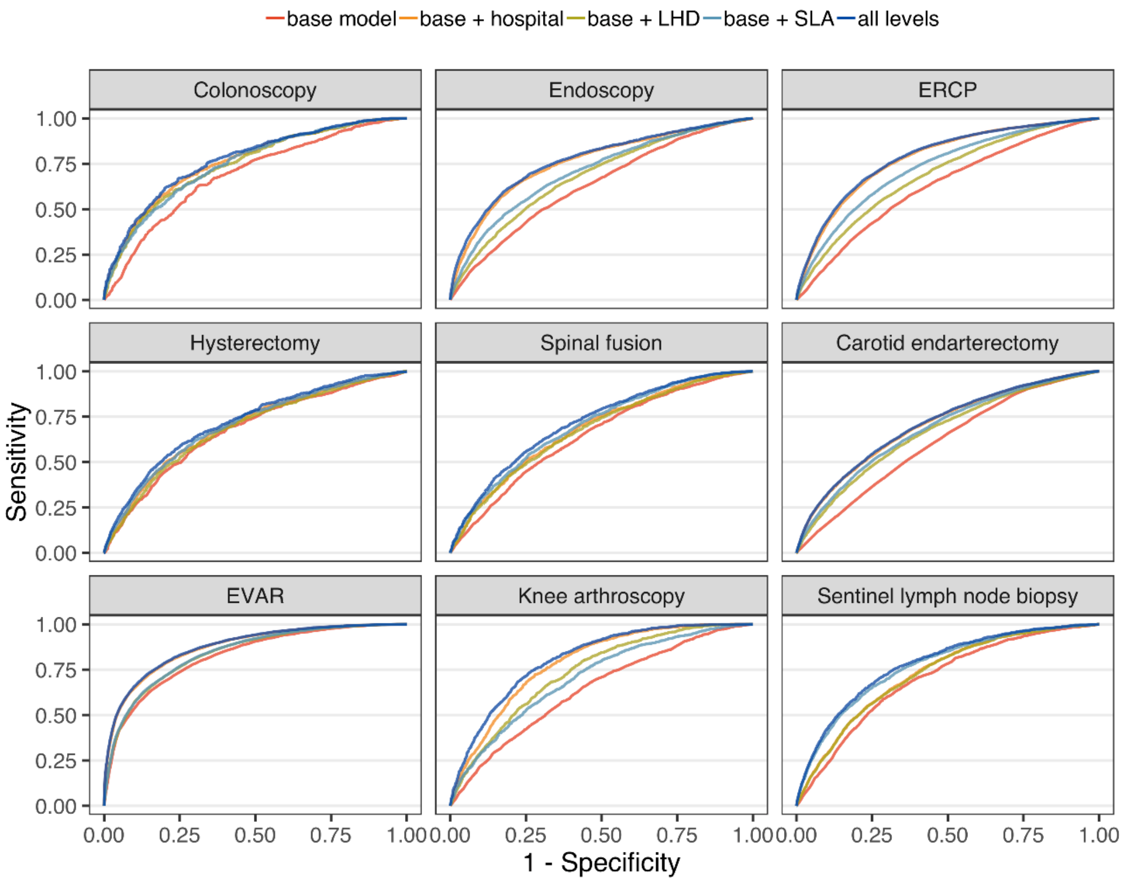

ERCP, endoscopic retrograde cholangiopancreatography. EVAR, endovascular repair of abdominal aortic aneurysm. LHD, Local Health District. SLA, Statistical Local Area.

Supplementary Figure 2: Proportion of residual variation at the hospital, Local Health District, and Statistical Local Area of residence levels, broader definitions of low-value care

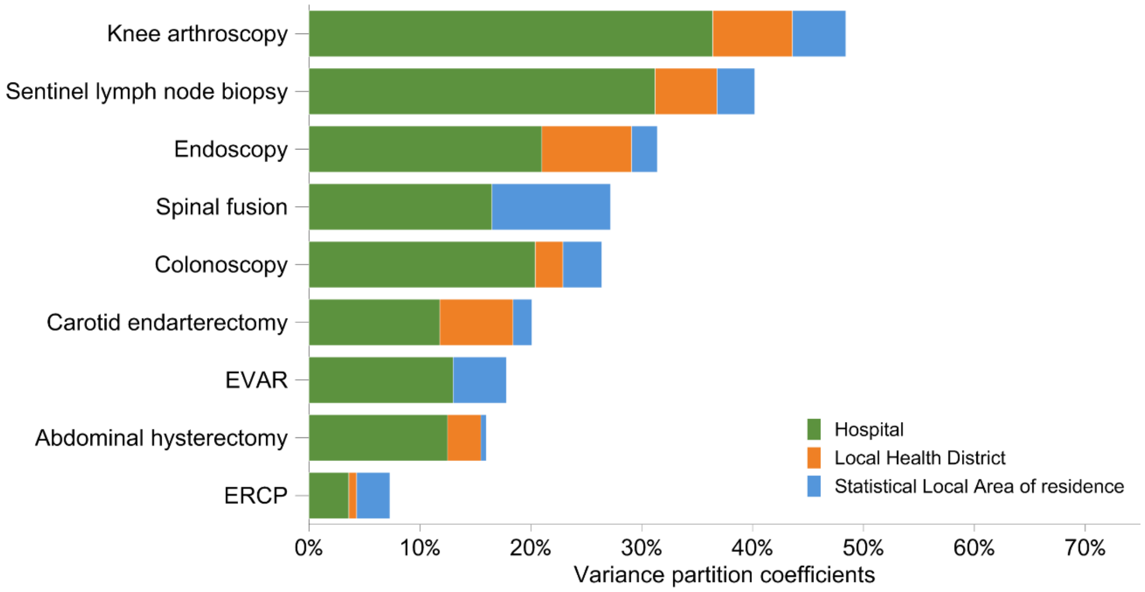

The total length of the bars shows the total proportion of variation explained by hospital, Local Health District (LHD), and Statistical Local Area. The remaining residual variation is attributable to unmeasured characteristics of the individual episode. LHD was not included in the models for EVAR and spinal fusion because of the small number of hospitals performing these procedures. ERCP, endoscopic retrograde cholangiopancreatography. EVAR, endovascular repair of abdominal aortic aneurysm.

Supplementary Figure 3: Median odds ratios, broader definitions of low-value care

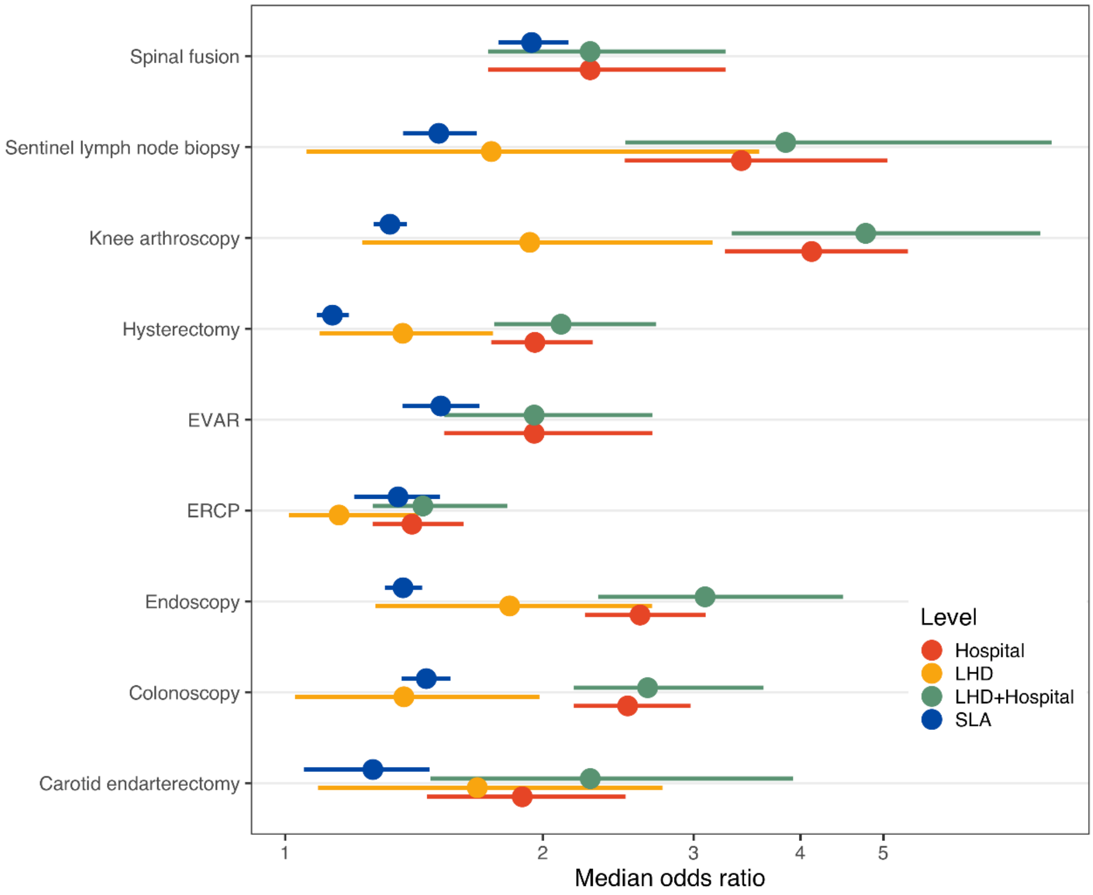

EVAR, endosvascular repair of aortic abdominal aneurysm. ERCP, endoscopic retrograde cholangiopancreatography. LHD, Local Health District. SLA, Statistical Local Area.
